# *De Novo* Synthesized Estradiol Protects against Methylmercury-Induced Neurotoxicity in Cultured Rat Hippocampal Slices

**DOI:** 10.1371/journal.pone.0055559

**Published:** 2013-02-06

**Authors:** Takeshi Yamazaki, Megumi Yamamoto, Yasuhiro Ishihara, Shota Komatsu, Eiji Munetsuna, Masahiro Onizaki, Atsuhiko Ishida, Suguru Kawato, Takao Mukuda

**Affiliations:** 1 Laboratory of Molecular Brain Science, Graduate School of Integrated Arts and Sciences, Hiroshima University, Higashi-Hiroshima, Japan; 2 Physiology Section, Department of Basic Medical Sciences, National Institute for Minamata Disease, Minamata, Kumamoto, Japan; 3 Department of Biophysics and Life Sciences, Graduate School of Arts and Sciences, The University of Tokyo at Komaba, Meguro, Tokyo, Japan; 4 Laboratory of Integrative Physiology, Graduate School of Integrated Arts and Sciences, Hiroshima University, Higashi-Hiroshima, Japan; University of Medicine & Dentistry of NJ - New Jersey Medical School, United States of America

## Abstract

**Background:**

Estrogen, a class of female sex steroids, is neuroprotective. Estrogen is synthesized in specific areas of the brain. There is a possibility that the *de novo* synthesized estrogen exerts protective effect in brain, although direct evidence for the neuroprotective function of brain-synthesized estrogen has not been clearly demonstrated. Methylmercury (MeHg) is a neurotoxin that induces neuronal degeneration in the central nervous system. The neurotoxicity of MeHg is region-specific, and the molecular mechanisms for the selective neurotoxicity are not well defined. In this study, the protective effect of *de novo* synthesized 17β-estradiol on MeHg-induced neurotoxicity in rat hippocampus was examined.

**Methodology/Principal Findings:**

Neurotoxic effect of MeHg on hippocampal organotypic slice culture was quantified by propidium iodide fluorescence imaging. Twenty-four-hour treatment of the slices with MeHg caused cell death in a dose-dependent manner. The toxicity of MeHg was attenuated by pre-treatment with exogenously added estradiol. The slices *de novo* synthesized estradiol. The estradiol synthesis was not affected by treatment with 1 µM MeHg. The toxicity of MeHg was enhanced by inhibition of *de novo* estradiol synthesis, and the enhancement of toxicity was recovered by the addition of exogenous estradiol. The neuroprotective effect of estradiol was inhibited by an estrogen receptor (ER) antagonist, and mimicked by pre-treatment of the slices with agonists for ERα and ERβ, indicating the neuroprotective effect was mediated by ERs.

**Conclusions/Significance:**

Hippocampus *de novo* synthesized estradiol protected hippocampal cells from MeHg-induced neurotoxicity via ERα- and ERβ-mediated pathways. The self-protective function of *de novo* synthesized estradiol might be one of the possible mechanisms for the selective sensitivity of the brain to MeHg toxicity.

## Introduction

Estrogen, a type of female sex steroid, is an important factor in the brain, where it has non-reproductive functions such as memory enhancement, neurotrophic action, and neuroprotection, as well as a role in reproductive behavior [1]. A protective effect of estrogen in Alzheimer’s disease, Parkinson’s disease and acute ischemic stroke has been demonstrated [2], [3], [4]. Estrogen is also neuroprotective against many insults, such as serum or oxygen-glucose deprivation, amyloid β peptide–induced toxicity, glutamate-induced excitotoxicity, and the toxicities of various chemicals, including MeHg [5], [6], [7], [8], [9], [10], [11].

Brain estrogen can be derived from peripheral steroidogenic organs via the blood stream, and from *de novo* synthesis at specific brain regions from steroid precursors or cholesterol [12]. Neurosteroids are synthesized from cholesterol in the central and peripheral nervous systems through mechanisms that are independent of peripheral steroidogenic organs, which include gonads and adrenal glands [12]. Estradiol concentrations were the same or higher in the newborn rat brain after removing the gonads and adrenals compared with sham-operated rats, indicating contribution of *de novo* estradiol synthesis as a neurosteroid [13]. The hippocampus is one of the regions that actively synthesize estradiol. The rat hippocampal cultured slices and dispersed cells synthesize 17β-estradiol as a neurosteroid [14], [15], [16]. In adult rats, the estradiol content of the hippocampus was 6 times higher than in plasma, indicating *de novo* synthesis of estradiol there [17].

Methylmercury (MeHg) is a hazardous pollutant and humans are exposed to MeHg mainly through consumption of fishes [18], [19]. Minamata disease, anthropogenic exposure to MeHg in Japan, and the MeHg poisoning in Iraq have established the toxicity of MeHg in the nervous system [20], [21], [22]. MeHg enters the central nervous system through the blood-brain barrier, and deposits of inorganic mercury are present diffusely in the brain. The lesions of Minamata disease are, however, localized in specific regions of the brain. Some parts of the cerebral and cerebellar cortices are affected more severely than other parts. There is no significant correlation between mercury deposition and lesion distribution [23], [24]. Molecular mechanisms for the selective susceptibility of each area of the brain to MeHg are not well defined, and they must be analyzed in order to understand the clinical symptoms [24].

The toxic effect of MeHg has been shown to be attenuated by exogenous estradiol in primary cultured rat granule cells [11]. In male mice, the administration of estradiol partially prevented motor activity deficits and the modification of cerebellar glutathione metabolism induced by MeHg [25]. Estrogen protects the brain through multiple mechanisms, both dependent on and independent of estrogen receptor (ER) activity. In the cultured granule cells, estradiol protected against MeHg toxicity by acting as an antioxidant. In these cells, estradiol functions the same way as J811, which is an artificial antioxidant that protects the cells without stimulation of estrogen receptors [11]. On the other hand, ER-mediated neuroprotection against excitotoxic glutamate and global ischemia has been shown to prevent neurodegeneration in cultured rat hippocampal neurons [26]. In the primary cultured neurons, both ERα and ERβ could be involved in the neuroprotection of exogenously added estradiol. Contribution of ERs on the neuroprotective function of estrogen against toxicity of MeHg was not yet defined well.

In this study, we examined the protective action of *de novo* synthesized estradiol against MeHg-induced cell death. To precisely determine estradiol synthesis in the hippocampus, rat hippocampal organotypic slice cultures were used for this study. The role of estrogen receptors in the protective effect of estradiol against MeHg-induced neurotoxicity was also examined.

## Materials and Methods

All procedures performed on animals were in accordance with the Fundamental Guidelines for Proper Conduct of Animal Experiments and Related Activities in Academic Research Institutions under the jurisdiction of the Ministry of Education, Culture, Sports, Science and Technology, Japan. The protocol was approved by the Animal Care and Use Committee of Hiroshima University, Hiroshima, Japan (The permission number is C10-32, which includes ethical approval.), and all efforts were made to minimize suffering.

### Rat Hippocampal Organotypic Slice Cultures

Rat hippocampal slices were prepared from 10- to 12-day-old male Wistar rats (SLC, Shizuoka, Japan) between 10:00 am-12:00 p.m. and were cultured as previously described [15], [27]. Approximately 12–15 slices of 0.3-mm thickness were obtained from one hippocampus. Five or 6 slices from the 8 hippocampi of 4 rats were randomly placed on one of the Millicell or Omnipore membranes (Millipore, Bedford, MA) that were laid on culture plates filled with culture medium [28]. The slices were pre-cultured at 37°C in a humidified incubator with 5% CO_2_ for 4 days. The medium consisted of 50% minimal essential medium, 25% Hank’s balanced salt solution (both from Sigma-Aldrich, MO, USA), and 25% horse serum (Invitrogen, Life Technologies Corp., Carlsbad, CA, USA) supplemented with penicillin-streptomycin solution (Sigma-Aldrich), followed by a day with serum-free medium consisting of 75% minimum essential medium and 25% Hanks’ balanced salt solution. After the pre-culture, MeHg was administered, and the slices were incubated for 24 h. Methylmercury chloride (Tokyo Chemical Industry, Tokyo, Japan) was dissolved in Dulbecco’s PBS (Sigma-Aldrich) with L-cysteine to 10 mM each to form MeHg-cysteine complexes (Hg:Cysteine = 1∶1). The MeHg-cysteine stock solution was filtered through a membrane filter (pore size 0.45 µm) and kept at −80°C. The stock solution was diluted with serum-free medium just prior to use. 17β-estradiol, 1,3,5-tris(4-hydroxyphenyl)-4-propyl-1H-pyrazole (PPT; Sigma-Aldrich), 2,3-bis(4-hydroxyphenyl) propionitrile (DPN; Sigma-Aldrich), ICI 182,780 (Tocris Bioscience, Bristol, UK), or ethanol (vehicle) was administered 2 or 6 h before the medium change. Letrozole (extracted from Femara tablet, Novartis, Basel, Switzerland) was added 24 h before the addition of MeHg.

### PI Uptake

Propidium iodide (PI) is a polar compound that only enters cells with damaged membranes and becomes brightly red fluorescent after binding nucleic acids [29], [30]. A concentration of 1 µM PI (Sigma-Aldrich) was added to the medium 22 h after the administration of MeHg. Two hours after the addition of PI, the slices were excited with a 540±25 nm light, and the emitted fluorescence was acquired at 605±55 nm on an inverted fluorescent microscope (BZ-9000, Keyence, Osaka, Japan). Fluorescence imaging was performed with Image J (http://rsbweb.nih.gov/ij/download.html). PI uptake as a neurodegeneration index was defined as the ratio of PI fluorescence intensity after the MeHg treatment to putative maximal values obtained after low-temperature exposure (4°C for 24 h).

### Quantitative RT-PCR

The determination of mRNA levels was performed according to our previous study [15]. Briefly, the total RNA was extracted from the 20 slices after a 24-h incubation with or without MeHg, and single-stranded cDNA was synthesized from the total RNA. Real-time PCR was performed using a LightCycler instrument (Roche Diagnostics, Basel, Switzerland) with primers described previously [15]. The amount of mRNA in MeHg-treated slices was calculated and compared to that in non-treated slices.

### Quantification of Estradiol

After 4 days of pre-culture with serum-containing medium, the slices were changed to serum-free medium, and the slices were incubated 48 h with MeHg, letrozole, or vehicle. To accurately measure the small amount of estradiol in the very fatty brain tissues, the steroid was extracted with an organic solvent, purified by solid column chromatography, and further purified by HPLC before analysis [31]. Estradiol in the 60 slices and the media was extracted and purified by a C18 mini-column and normal-phase HPLC as described previously [15]. The purified estradiol was quantified using an EIA kit (Cayman Chemical, Ann Arbor, MI, USA). To confirm that we were detecting chemically distinct 17β-estradiol in the EIA, some samples were also analyzed by liquid chromatography-tandem mass spectrometry (LC-MS/MS) at Asuka Pharmamedical Co. Ltd. (Kawasaki, Japan).

### Statistical Analyses

All frequencies were analyzed by one-way ANOVA for significance, and multiple comparisons using the Holm-Bonferroni method were performed to detect significant differences. *P*<0.05 was considered significant.

## Results

### Pre-culture Period

**Figure 1:**
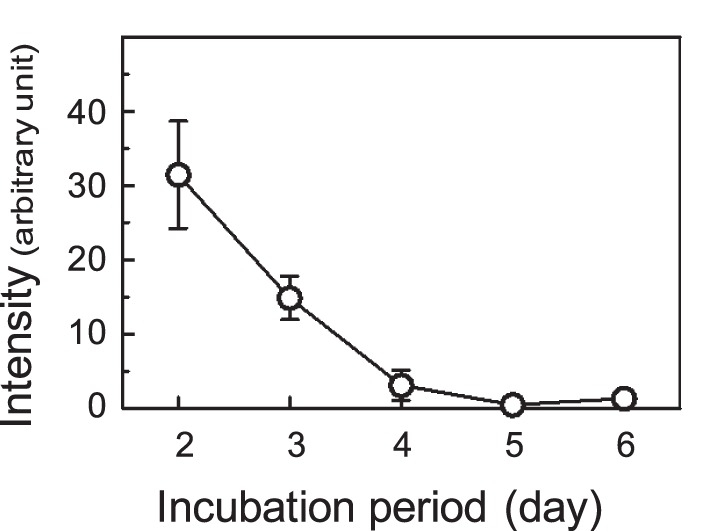
The PI fluorescence intensity of cultured slices. The hippocampal slices were cultured for 6 days with serum-containing medium. The intensity of PI fluorescence of whole slices is indicated. The values shown are the means of 9 slices in three different cultures, and the error bars indicate the standard error (SE).

First, we determined the pre-culture period for the detection of tissue cell death in the rat hippocampal organotypic slice cultures. The maximum level of estradiol synthesis is typically obtained from slices after a short pre-culture time [15]. In this experiment, cell death in the cultured slices was determined by PI staining. PI uptake is a good index for damaged cells in the slices [27], [32]. The fluorescence of PI staining of the slices was high at the onset of the culture, decreased to a negligible level after 5 days in culture (Fig. 1). We therefore used the slices after a 5-day pre-culture, 4 days with serum-containing medium and a day with serum-free to ensure both the high steroidogenic activity and precise analysis of tissue cell death under a low PI-staining background.

### The Effects of MeHg on Hippocampal Slice Cultures

**Figure 2:**
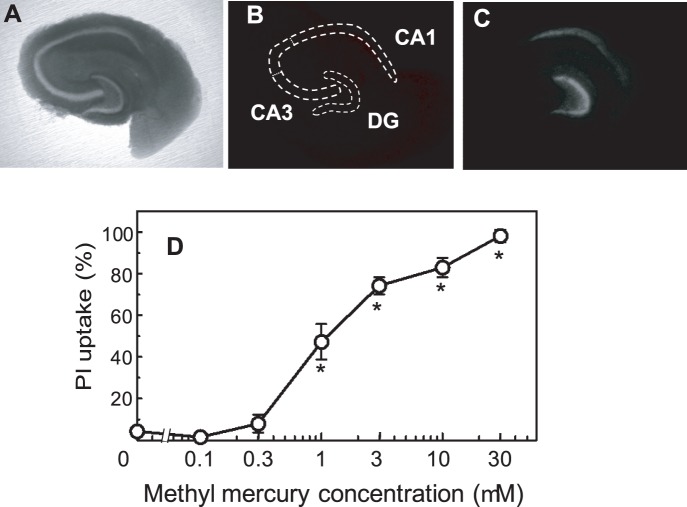
MeHg-induced cell death as assessed by PI uptake. Bright field (A) and PI fluorescence (B) images after 5 days of pre-culture and PI fluorescence after subsequent incubation with 1 µM MeHg for 24 h (C) of the cultured hippocampal slices. Neuronal cell dense regions of dentate gyrus (DG), and CA1 and CA3 regions in Ammon’s horn are traced on (A) and superimposed on (B) as dotted lines. The concentration dependency of MeHg on PI uptake is shown in (D). The PI uptake is expressed as a percentage of total fluorescence in the neuronal cell dense region (inside the dotted lines in B) after the slices were kept at 4°C for 24 h. The values are the means of 15–21 slices in 5 different cultures, and the error bars indicate the SE. *, *P*<0.05, vs. MeHg-treated group, post hoc *t*-test with Holm-Bonferroni method after one-way ANOVA.

The hippocampal neuronal cell dense regions were easily detected in bright field images of the hippocampal slices (Fig. 2A). After a 5-day pre-culture, the PI fluorescence was barely detectable in the slices (Fig. 2B). After the subsequent incubation with 1 µM MeHg, the cells in the neuronal cell dense regions were stained by PI, indicating selective vulnerability of the cells in the regions. As shown in Fig. 2C, the dentate gyrus (DG) was stained most significantly. CA1 and CA3 regions in Ammon's horn were also stained. This result is consistent with a previous observation that MeHg-exposure during the perinatal period acutely induced cell death in the rat hippocampus, especially in the dentate gyrus [33]. The MeHg dose dependence of the PI uptake in the Ammon's horn and dentate gyrus is shown in Fig. 2D. After a 24-h treatment with 1 µM MeHg, the PI uptake was approximately 50%, which was suitable for the evaluation of neurotoxic effects. We used 1 µM of MeHg for subsequent experiments.

### The Effects of Exogenously Added Estradiol on MeHg Neurotoxicity

**Figure 3:**
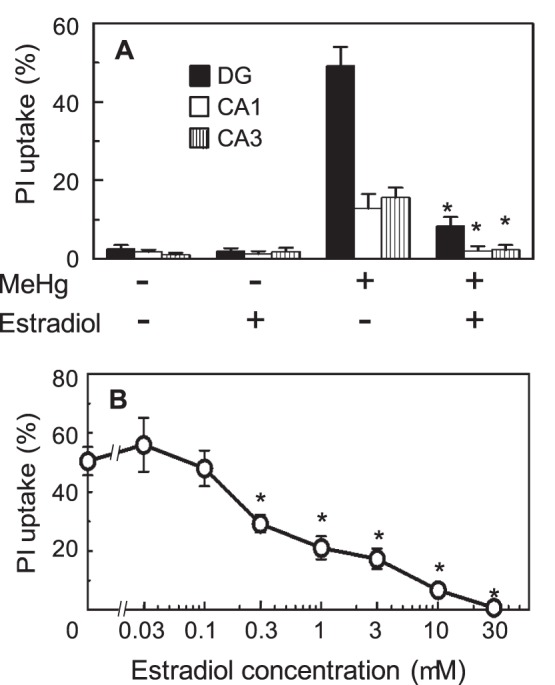
The effect of exogenously added estradiol on MeHg-induced cell death. The PI uptake of the hippocampal slices was analyzed individually in the neuronal cell dense regions, dentate gyrus (DG), and CA1 and CA3 regions in Ammon’s horn, which are shown in Fig. 2B (A). Slices were incubated with vehicle or 10 µM estradiol for 26 h, and/or for 24 h with 1 µM MeHg. MeHg was added 2 h after administration of estradiol. The values are the means of 14–21 slices in 5 different cultures, and the error bars indicate the SE. *, *P*<0.05 vs. MeHg-treated group, post hoc *t*-test with the Holm-Bonferroni method after one-way ANOVA. The concentration dependency of the protective effect of estradiol on the PI uptake of the neuronal cell dense region of the 1 µM MeHg-treated slice is shown in (B). The values are the mean of 14–23 slices in 4 different cultures, and the error bars indicate the SE. *, *P*<0.05 vs. MeHg-treated group, post hoc *t*-test with Holm-Bonferroni method after one-way ANOVA.

Incubation of slices with 10 µM estradiol for 26 h had no effect on PI uptake in the DG, or CA1 and CA2 in Ammon’s horn of the hippocampus (Fig. 3A). PI uptake was increased by a 24 h treatment with 1 µM MeHg. Pre-treatment of the slices with 10 µM estradiol 2 h before the addition of 1 µM MeHg decreased PI uptake (Fig. 3A). The neuroprotective effects of estradiol on these three regions were similar; therefore, the PI uptake was analyzed on the total neuronal cell dense region for subsequent experiments. The dose dependence of the effects of estradiol in protecting against MeHg-induced neurotoxicity is shown in Fig. 3B. PI staining of the neuronal cell dense region in the hippocampus was completely blocked by pre-treatment with 30 µM estradiol.

### The Effects of MeHg on mRNA and Estradiol Concentration in Hippocampal Slices

MeHg has been shown to affect steroidogenic activity in the adrenal gland and gonads in various animals [10]. In mammals, brain injury induces estrogen synthesis via the rapid transcription and translation of CYP19 (P450arom) in astroglia [34]. Transcription of steroidogenic acute regulatory protein (StAR), CYP11A1 (P450scc), ERα, and ERβ was also upregulated by injury in various brain regions [35]. Therefore, we analyzed the effect of MeHg on the transcript levels of these proteins and the other steroidogenic enzymes, 3β-hydroxysteroid dehydrogenase/Δ^5^–Δ^4^ isomerase (3β-HSD) type-I and II, CYP17 (P450_17α_)_,_ 17β-hydroxysteroid dehydrogenase (17β-HSD) type-III and IV, and adrenal 4-binding protein/steroidogenic factor-1 (AD4BP) in the hippocampal slices by real-time RT-PCR. No significant changes were observed in mRNA levels for these proteins after incubation with or without 1 µM MeHg (Fig. S1).

The estradiol content of the hippocampal slices and medium was determined by EIA. The accuracy of the EIA data was cross-checked by LC-MS/MS. Estradiol contents of the slices and medium after the 48-h incubation with or without 1 µM MeHg was 48.8±6.1 and 54.9±11.2 fmol per mg protein, respectively (n = 3). Incubation with MeHg had no significant effect on the estradiol content of the slices and the medium.

We therefore conclude that estradiol synthesis in the slices was not affected by treatment with 1 µM MeHg under these experimental conditions.

### The Effects of an Estrogen Synthesis Inhibitor and an ER Antagonist

**Figure 4:**
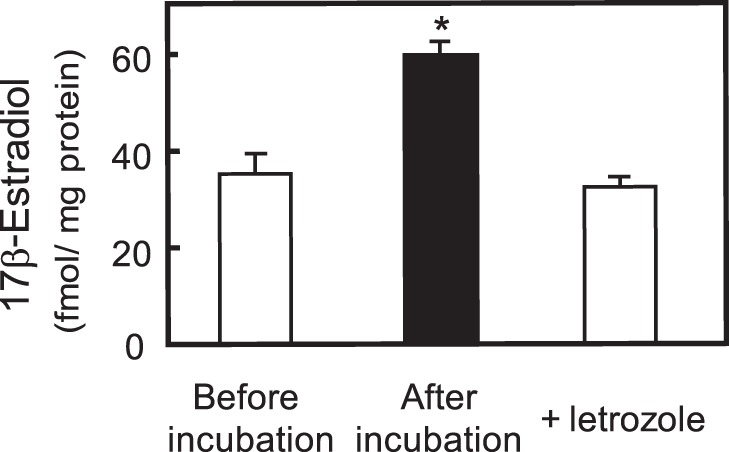
The inhibition of estradiol synthesis by letrozole. Estradiol was extracted from slices and medium before and after a 48-h incubation with or without letrozole. Letrozole was extracted from Femara tablets. The levels of extracted 17β-estradiol were quantified as described in the Materials and Methods section. The values are the means of 3 separate experiments, and the error bars indicate the SE. *, *P*<0.05, vs. non-treated group, *t*-test.

Letrozole is a specific inhibitor for P450arom. The treatment of cultured hippocampal neurons and slices with letrozole induces a decrease in hippocampal estradiol synthesis [36]. The estradiol content of hippocampal slices after the 4-day pre-culture was 35.2±4.3 fmol per mg protein (Fig. 4). Then, the slices were incubated with serum-free medium for 48 h. Estradiol content was increased to 59.8±3.0 fmol per mg protein after the incubation (Fig. 4). The increment of estradiol, 25 fmol per mg protein, indicated *de novo* synthesis in the hippocampus. After the incubation with letrozole for 48 h, the estradiol content, 32.6±1.7 fmol per mg protein, was not significantly changed from that of before incubation (Fig. 4). Letrozole treatment completely inhibited *de novo* estradiol synthesis in this condition.

**Figure 5:**
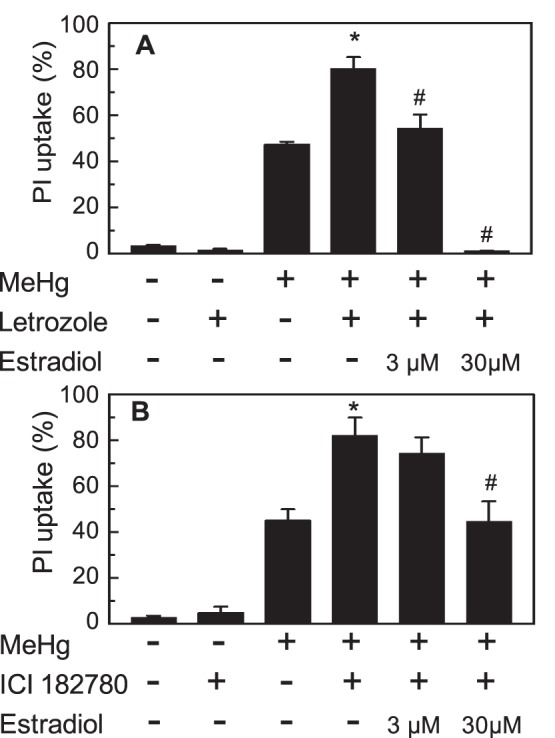
The effects of letrozole and ICI on MeHg-induced cell death. Levels of PI uptake in the neuronal cell dense region of the slices after a 48-h treatment with vehicle or letrozole and/or 24-h treatment with 1 µM MeHg are shown in A. Levels of PI uptake after a 30-h treatment with vehicle or 100 µM ICI 182,780 and/or 24-h treatment with 1 µM MeHg are shown in B. MeHg was added 24 or 6 h after the administration of Letrozole or ICI 182,780, respectively. Some wells were given the indicated concentrations of estradiol 2 h before the addition of MeHg. The values are the means of 16–21 slices in 5 different cultures or 13–16 slices in 4 different cultures for A or B, respectively, and the error bars indicate the SE. *, *P*<0.05, vs. the MeHg-treated group, and #, *P*<0.05, vs. MeHg and letrozole- or ICI-treated group, post hoc *t*-test with the Holm-Bonferroni method after one-way ANOVA.

Treatment of the slices with letrozole for 48 h induced no change in PI uptake, indicating that letrozole was not neurotoxic by itself (Fig. 5A). After the slices were incubated for 24 h with letrozole followed by 24 h with 1 µM MeHg and letrozole, the PI uptake was approximately 80%, which was 30% higher than the slices treated with MeHg only (Fig. 5A). The increase in PI uptake was reversed by the co-addition of 3 µM estradiol 2 h before the administration of MeHg. These data indicate that the hippocampus-synthesized estradiol attenuated the toxic effects of MeHg. The co-addition of 30 µM estradiol blocked PI uptake almost completely, similar to the data shown in figure 3B, indicating no effect of letrozole on the neuroprotective effect of externally added estradiol against MeHg.

ICI 182,780 is an antagonist both for ERα and ERβ [37]. Treatment of the slices with 100 µM ICI 182,780 for 30 h had no effect on the PI uptake of the slices, indicating that ICI 182,780 was not neurotoxic by itself. In the presence of ICI 182,780, PI uptake after 1 µM MeHg treatment was increased to 82%, the same level as that of the letrozole-treated slices (Fig. 5A, B). These data indicate that the protective function of *de novo* synthesized estradiol might be mediated by ERs in the hippocampal slices.

ICI 182,780 also blocked the protective effect of exogenously added estradiol. Pre-treatment of the slices with 3 µM estradiol had no significant effect on MeHg-induced cell death in the presence of ICI 182,780 (Fig. 5B). Interestingly, PI uptake was significantly decreased by pre-treatment with 30 µM estradiol in the presence of ICI 182,780, although the protective effect was far less than in the absence of the antagonist (Figs. 3B, 5A, and 5B).

### The Effect of Estrogen Receptor Agonists on the Neurotoxicity of MeHg

**Figure 6:**
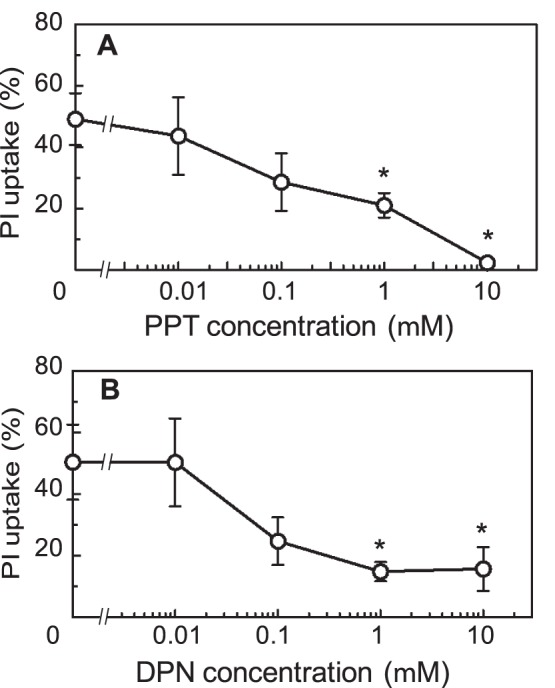
The effects of estrogen receptor agonists on MeHg-induced cell death. The PI uptake of neuronal cell dense region of the slices was determined after a 24-h treatment with 1 µM MeHg in the presence of various concentrations of PPT (A) or DPN (B). These agonists were administered 2 h before the addition of MeHg. The values are the means of 16–22 slices in four different cultures, and the error bars indicate the SE. *, *P*<0.05, vs. MeHg-treated group, post hoc *t*-test with Holm-Bonferroni method after one-way ANOVA.

PPT and DPN are specific agonists for ERα and ERβ, respectively [37]. To evaluate the contribution of ERs to the protective effect, these agonists were added 2 h before the slices were treated with 1 µM MeHg. PI uptake in the neuronal cell dense region was decreased by the pre-addition of these agonists in a dose-dependent manner. A significant protective effect was observed at PPT or DPN concentrations higher than 1 µM (Fig. 6). These data indicate that both ERα- and ERβ-dependent pathways might mediate neuroprotection against MeHg.

## Discussion

In this study, the protective effects of hippocampal *de novo* synthesized estradiol against MeHg-induced tissue cell death were demonstrated by the following results: (1) exogenously added estradiol protected rat hippocampal slices from the toxic effect of MeHg (Fig. 3), (2) the slices synthesized estradiol (Fig. 4), (3) *de novo* estradiol synthesis was not affected by 1 µM MeHg, and (4) letrozole inhibited *de novo* estradiol synthesis and increased the neurotoxicity of MeHg (Fig. 4, 5A). Enhancement of the neurotoxic effect of MeHg by letrozole treatment was not caused by accumulation of estradiol precursors, because co-addition of exogenous estradiol with letrozole restored the protective effect (Fig. 5A).

The protective effect of *de novo* synthesized estradiol in the slices was abrogated by ICI 182,780, an ER antagonist, and was not counteracted by the addition of exogenous estradiol in the presence of the antagonist (Fig. 5B). The protective effect of estradiol was mimicked by the administration of specific agonists for ERα or ERβ (Fig. 6). The rat hippocampus possesses substantial amounts of ERα and ERβ mRNA [15]. Taken together, we concluded that the protective effect of *de novo* synthesized estradiol against neurotoxicity of MeHg was mediated by both ERα and ERβ.

ERα- and ERβ-mediated neuroprotection by estradiol could be induced by classical genomic effects and/or by non-classical mechanisms [5], [8], [34]. In the classical genomic effect, ligand binding to ERα and ERβ induces dimerization of these receptors, which form either homodimers or heterodimers, to bind estrogen-responsive elements on target genes. The target genes for estradiol-induced neuroprotection have been reported to be neurotrophic factors as well as their receptors, apoptosis-related genes, and genes that modulate cellular architecture. In the non-classical mechanisms of ERs, various signal transduction pathways are activated by estradiol, including intracellular Ca^2+^, extracellular-signal regulated kinases 1 and 2 (ERK1/2), and G-protein-coupled receptors, etc. The neurotoxicity of MeHg may be mediated by molecular mechanisms of a variety of cellular functions, including intracellular Ca^2+^ and glutathione homeostasis, maintenance of the mitochondrial membrane potential, assembly and disassembly of microtubules, and activity of the Na^+^/K^+^ pump [38]. MeHg exposure generates reactive oxygen species and radicals in neurons, which may induce cell damage [39], [40], [41]. The precise molecular mechanism of the neuroprotective effect of estradiol in rat hippocampal slices is currently under investigation in our laboratory.

Although the protective effects of *de novo* synthesized and lower concentrations of exogenous estradiol were mediated by the ERs, 30 µM exogenous estradiol partly protected hippocampal cells from the toxic effects of MeHg in the presence of ICI 182,780 (Fig. 5A). This high concentration of estradiol might protect against cell death without activation of the estrogen receptor system. Estradiol itself is a neuroprotective antioxidant at concentrations higher than a few micromolar [5], [8]. Indeed, the neurotoxic effect of MeHg on cultured cerebellar granule cells was abrogated by the antioxidant function of 10 µM estradiol [11]. On the other hand, multiple molecular mechanisms for receptor-independent neuroprotection by estradiol have been proposed. The indirect genomic mechanism requires the rapid activation of mitogen-activated protein kinases and Akt signaling pathways and estrogen-induced activation of a transcription factor cAMP-responsive element binding protein [5], [8]. The receptor-independent mechanisms of neuroprotection by estradiol are also under examination in our laboratory.

In this *in vitro* study, estradiol synthesis in the hippocampal slices was monitored by determination of actual estradiol contents in the slice to evaluate the significance of *de novo* estradiol synthesis on the neuronal self-protection. Our results are consistent with an *in vivo* study; intracerebral administration of fadrozole, another P450arom inhibitor, enhanced kainic acid-induced neurodegeneration in the hippocampus of the male rat, although *de novo* estradiol synthesis was not quantified in the *in vivo* brain [42]. Both their study and ours are, however, lacking in information about the source of the *de novo* estradiol synthesis. In our *in vitro* experiment, slices were cultured in serum-free medium, which did not contain detectable steroids. We have previously reported that stimulation of *de novo* estradiol synthesis in the slices by 9-*cis*-retinoic acid is concomitant with increases in *de novo* testosterone synthesis and the mRNA level of P450_17α_, indicating that the precursors of these steroids are located upstream of P450_17α_ activity, such as pregnenolone, progesterone, and/or cholesterol [15]. In *in vivo* hippocampus, estradiol might be synthesized from precursors from both inside and outside the brain. Plasma testosterone has high blood-brain barrier permeability and may be a substrate for P450arom [43]. Decrease in plasma testosterone by castration, however, only affected hippocampal estradiol contents very slightly. Estradiol content in castrated rat hippocampus was 83% of that in sham-operated rats, although plasma testosterone concentration was decreased to one-seventieth [31]. The *in vivo* hippocampus might synthesize estradiol also from substrates other than plasma testosterone, such as pregnenolone, progesterone and/or cholesterol.

Brain estradiol can be derived from peripheral steroidogenic organs via the blood stream, in addition to *de novo* synthesis. Plasma estradiol may distribute through the entire brain because estradiol has high blood-brain barrier permeability [43]. The *de novo* estradiol synthesis is, however, limited to discrete regions and varies by developmental stage and animal species [12]. The self-protective effect of *de novo* synthesized estradiol may be region-specific in the brain and vary by developmental stage and animal species. Indeed, the estradiol content and the mRNA level for P450arom in the cerebral cortex was about one-half and one-fifth, respectively, of those in the hippocampus of 10-day-old rats (unpublished result). This result is consistent with the observation that the neuronal damage in the cerebral cortex was higher than that in the hippocampus of 15-day-old rats after administration of MeHg [44]. In the mouse brain, MeHg induced neuropathological changes in the cerebral cortex but not in the hippocampus, despite a similar accumulation of mercury [45]. The pathological changes observed in Minamata disease occur predominantly in specific areas, including the calcarine region, the postcentral and precentral gyri, and the temporal transverse gyrus [24]. Mercury was, however, detected over a wide area of the brain including the thalamus, pallidum and caudate nucleus with Minamata disease following a 26 year clinical course after the first severe attack in 1956 [46]. Although *de novo* estrogen synthesis in local areas of human brain is not yet well analyzed, mRNA for P450arom was lower in cerebellum than thalamus [47]. To understand the molecular mechanism for the region specific lesions in different brain areas, the self-protective effect of *de novo* synthesized estradiol is worth considering as one of the possible factors.

## Supporting Information

Figure S1 **The effect of MeHg on mRNA levels in the slices.** The slices were incubated with or without 1 µM MeHg for 24 h. The amounts of mRNA were determined by real-time RT-PCR as described in the Materials and Methods section. The amounts of mRNA in MeHg-treated slices were given as the values relative to that in the non-treated slices. The values are the means of 3 separate experiments, and the error bars indicate the SE.
(EPS)
