# Correction: *De Novo* Synthesized Estradiol Protects against Methylmercury-Induced Neurotoxicity in Cultured Rat Hippocampal Slices

**DOI:** 10.1371/annotation/52376e1c-1a2d-44af-a129-849345da78a0

**Published:** 2013-04-22

**Authors:** Takeshi Yamazaki, Megumi Yamamoto, Yasuhiro Ishihara, Shota Komatsu, Eiji Munetsuna, Masahiro Onizaki, Atsuhiko Ishida, Suguru Kawato, Takao Mukuda

In the x-axes of Figures 2, 3, and 6, the Greek letter "**μ**" was incorrectly replaced by the letter "**m**". The correct Figure 2 can be viewed here: 

**Figure:**
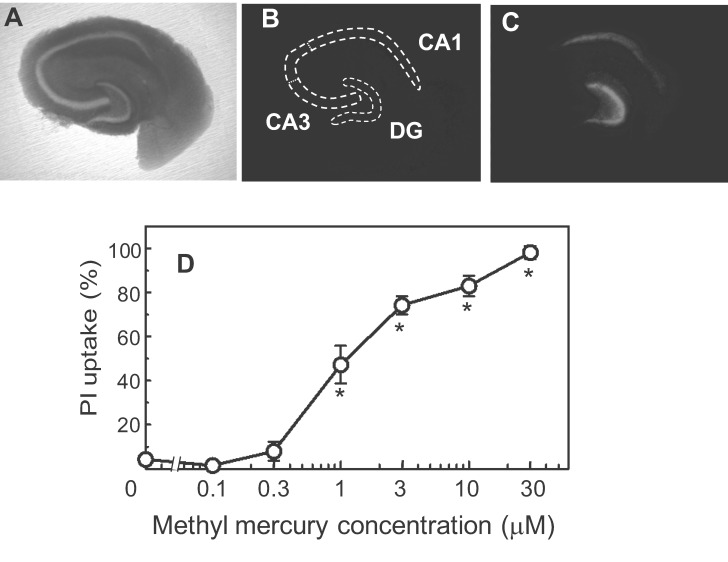


. The correct Figure 3 can be viewed here: 

**Figure:**
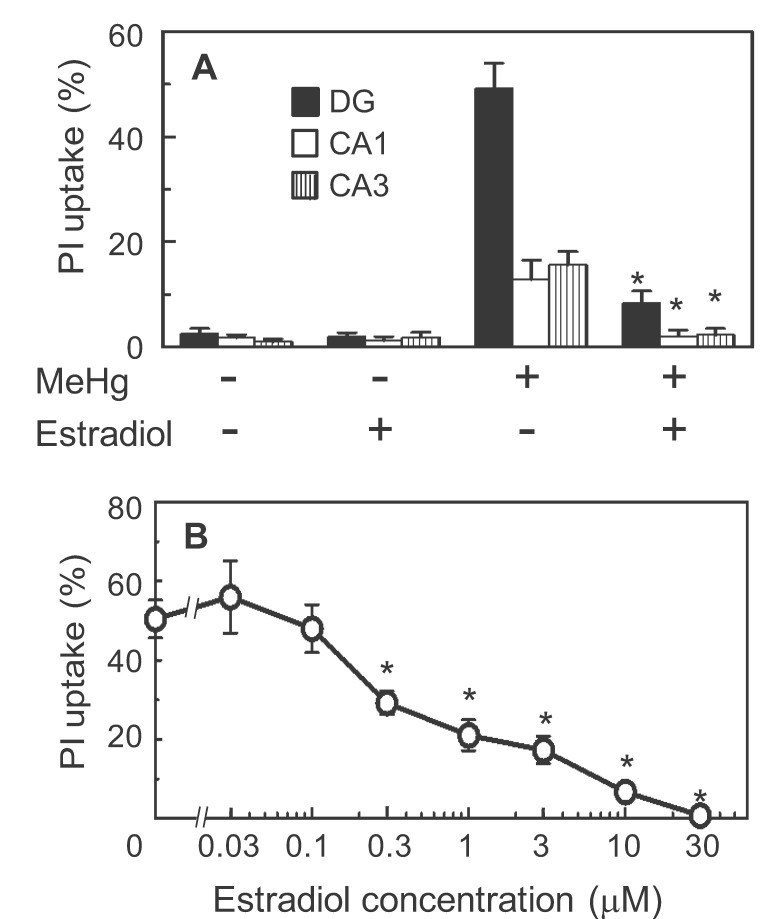


. The correct Figure 6 can be viewed here: 

**Figure:**
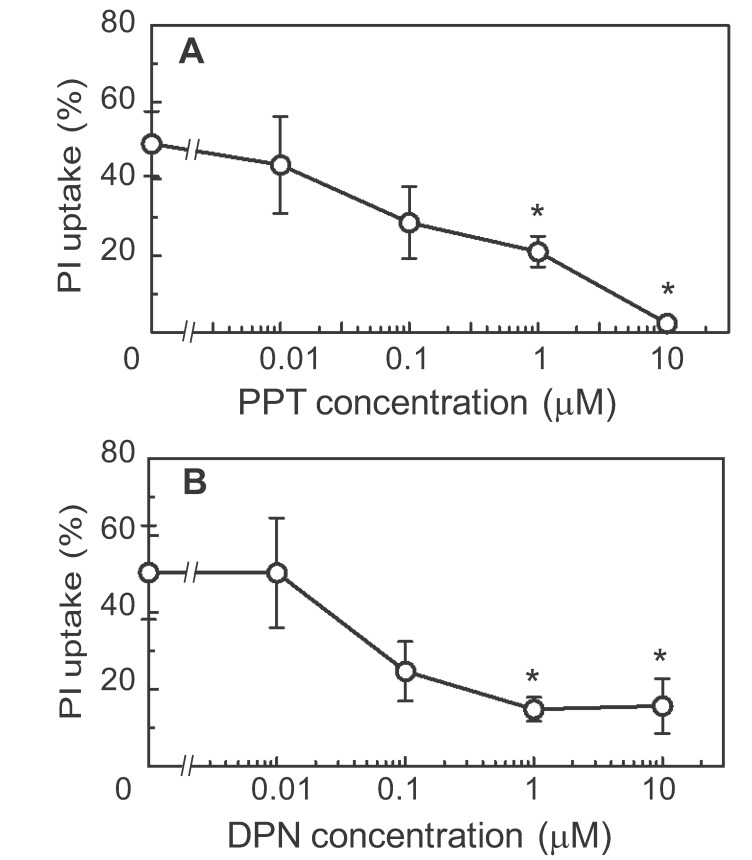


. 

